# Plantains and Bananas

**Published:** 1888-12

**Authors:** 


### PLANTAINS AND BANANAS.

The banana is much softer and more delicately flavored than the plantain. The fruit when ripe contains 74 per cent. water ; of the twenty-six remaining parts, twenty are sugar and two gluten or flesh forming substance. Like rice, it is not by itself a perfect food, but requiring the addition of some more nitrogenous material, as pulse or lean meat. When dried and converted into meal it is less nutritious than the meal of the grain mentioned. In tropical countries it is, nevertheless, a most valuable food, and is so extensively consumed as to take the place of our cereal grains as the ordinary article of diet. About six and a half pounds of the fruit, or two pounds of the dry meal, with one-quarter pound of salt meat or fish, form in tropical America the daily allowance for a laborer. The pulp of the plantain is squeezed through a fine sieve and formed into loaves, which, when dried, will keep a long time. The unripe fruit often becomes the staff of life ; it is dried in the oven and is eaten in this state, and will keep such a length of time that natives carry it with them when proceeding on journeys. While unripe the fruit is filled with starch, and when dried has a resemblance to bread both in taste and composition. The loaves made by the ripened pulp are saccharine and not farinaceous.

There are many instances of men subsisting entirely upon bananas, who have been deserters from ships in tropical climes. Mr. Stanley, in his book, "Across the Dark Continent," frequently mentions bananas as one of the principal articles of food for his party, and as one of the most important crops of the natives. He states that "villages and banana fields were always found together." Also, that in the dwarf land bananas as long as his arm and plaintains as long as the dwarfs (just one yard), were sufficient food for a man one day. He says, besides, that he was "compelled for weeks to subsist on green bananas, cassava and sugarless tea," and a scant supply at that. At the place on the Congo he purchased "ten bananas, thirteen inches in length by three inches in diameter."

In British Guiana and other tropical countries bananas are largely used as the food of infants, children and invalids. They are of higher alimentary value than such preparations as sago or arrow root, because containing a larger proportion of starch and a certain percentage of nitrogenous matter.

The name of the plantain is derived from the Spanish name, plantano, by which this plant is known in foreign countries, the name banana ever being used. It forms a spurious kind of stem five to thirty-five feet in height, by the sheathing bases of its leaves—those on the largest varieties measuring over ten feet long and two feet wide. The stem sends out these large leaves from the centre of the top until it arrives at maturity, when a smaller leaf appears, which indicates that it will fruit. The fruit stem then sends out a large purple bud shaped like an ear of corn in its husk. The fruit grows on this stem in "hands" containing from eight to fourteen bananas, and there are about eight to ten of these hands constituting the "bunch," which often weighs one hundred pounds and averages forty pounds. These hands are somewhat spirally placed on the stem. The evolution of the banana on the stem is peculiar and very interesting. First comes the large purple bud or cone already mentioned. This is about twelve inches long and three inches in diameter, having a number of leaves. Secondly, under the first leaf on this cone in due time come from eight to fourteen blossoms. Thirdly, these blossoms in their turn after awhile grow little bananas at their bases in the same way as apples with us grow from their blossoms. Finally, when these bananas have grown about two inches long, the leaf that covers them dries and rolls up and then falls off, and the first "hand" of bananas is revealed, looking quite cute and curious. The second and succeeding hands are formed in the same way, but meanwhile the cone keeps growing in length and the stem in thickness, thus giving space between the "hands" for the fruit when fully grown, and strength to the stem to sustain the great weight of the completed bunch. The fruit of a single tree averages from thirty to forty pounds, and often seventy or one hundred pounds.

The banana tree bears but one bunch of fruit before it dies down, when from two to ten trees spring up around it.

The banana is eaten in many ways, but principally raw when ripe. A certain coarse banana, which is not agreeable to eat raw, is peeled, sliced and fried in hot lard, and is a delicious vegetable for the table. Those who purchase green bananas by the bunch to ripen in the store closet, will find this an excellent and acceptable way to cook some of them.

The fibre of the banana tree is worth more than its fruit, which cost but fifteen cents a bunch in its native clime ; but this fibre in many countries is permitted to go to waste. In Central America this fibre, with only the preparation of drying, is made into shoestrings and cords for all uses. It is divided into threads of silken fineness extending the length of the body of the tree, which shoots up from ten to fifteen feet without a branch.

Within the last three years the imports of bananas at the port of New York alone, has doubled. Three million bunches are brought here annually, and nearly all of them in steamships loaded only with this fruit. Formerly they were brought here in "Fruiters," or fast sailing ships. About one-half of the imports come from the port of Baracoa, Cuba, but the Island of Jamaica is increasing her exports of this fruit so rapidly that now they amount to $200,000 more than what is realized on sugar. In August of this present year there were ten steamships loaded in one week with 120,000 bunches, valued at $60,000, for Northern ports of this country. It is estimated that the increased receipts of bananas per year is 40 per cent.

Formerly New York was the only port at which the banana was received, but since the trade has increased so extensively Boston, Philadelphia and Baltimore are now importing the fruit.

The yellow banana importations are 40 per cent. more than those of the red species. The sale of the red banana is confined to New York, the Eastern States and some portions of Canada. The yellow fruit is preferred on account of its superior flavor, and because it is more profitable to handle. In a bunch of yellow fruit there are just about twice as many bananas as in a bunch of the red variety.

The opinions of physicians in regard to bananas as food for the sick have altered in about the same degree as their ideas concerning milk. At one time the latter was supposed to be "feverish" and was denied a typhoid patient Now it is their chief subsistence. Bananas served cold from the ice are very acceptable to the sick, and are now allowed by most of the medical faculty.

